# Differential Expression of 26S Proteasome Subunits and Functional Activity during Neonatal Development

**DOI:** 10.3390/biom4030812

**Published:** 2014-08-29

**Authors:** Erika C. Claud, Julie A. K. McDonald, Shu-Mei He, Yueyue Yu, Lily Duong, Jun Sun, Elaine O. Petrof

**Affiliations:** 1 Departments of Pediatrics and Medicine, University of Chicago, Chicago, IL 60611, USA; E-Mails: eclaud@peds.bsd.uchicago.edu (E.C.C.); yyu@peds.bsd.uchicago.edu (Y.Y.); 2 Gastrointestinal Diseases Research Unit, Department of Medicine, Queen’s University, Kingston, ON K7L 2V7, Canada; E-Mails: jm13@queensu.ca (J.A.K.M.); smh6@queensu.ca (S.-M.H.); 9ld27@queensu.ca (L.D.); 3 Department of Biochemistry, Rush University, Chicago, IL 60612, USA; E-Mail: Jun_Sun@rush.edu

**Keywords:** proteasome, immunoproteasome, expression, catalytic, weaning

## Abstract

Proteasomes regulate many essential cellular processes by degrading intracellular proteins. While aging is known to be associated with dysfunction of the proteasome, there are few reports detailing activity and function of proteasomes in the early stages of life. To elucidate the function and development of mammalian proteasomes, 26S proteasomes were affinity-purified from rat intestine, spleen and liver. The developmental expression of core, regulatory and immunoproteasome subunits was analyzed by immunoblotting and reverse-transcriptase PCR of mRNA subunits, and proteasome catalytic function was determined by fluorogenic enzymatic assays. The expression of core (β2, β5, α7 and β1) and regulatory (Rpt5) subunits was found to be present at low levels at birth and increased over time particularly at weaning. In contrast, while gradual developmental progression of proteasome structure was also seen with the immunoproteasome subunits (β1i, β5i, and β2i), these were not present at birth. Our studies demonstrate a developmental pattern to 26S proteasome activity and subunit expression, with low levels of core proteasome components and absence of immunoproteasomes at birth followed by increases at later developmental stages. This correlates with findings from other studies of a developmental hyporesponsiveness of the adaptive immune system to allow establishment of microbial colonization immediately after birth.

## 1. Introduction

The intestine is a key organ for digestion and immune activity, however it is a relatively dormant organ for the fetus. Thus, the transition from *in utero* to *ex-utero* life involves many critical alterations. These include altered blood flow and growth to allow for digestion, but also a progression in immune response to allow for microbial colonization, and appropriate immune responses to food and microbial antigens [1,2].

Important to these processes are proteasomes, which are multi-subunit proteases that play a critical role in degrading misfolded, damaged, or oxidized cellular proteins [3] and in the regulation of essential cellular processes (e.g., cell cycle, inflammatory response, immune response) [4,5]. The proteasome system is structurally and functionally diverse, sharing a core structural design (20S) with varying functions and subunit compositions that can vary by tissue [6,7].

The 26S and 20S proteasomes are both functional proteasomes that cleave proteins. The 26S proteasome is a barrel-shaped protein complex, comprised of a 20S “core” that has two identical outer rings of α1–α7 structural subunits and two identical inner rings of β1–β7 subunits, as well as two 19S regulatory “lids” on either end of the core 20S catalytic barrel [8]. The regulatory 19S subunits are composed of a base containing a hexameric ring of six AAA-ATPases (Rpt1-6) which are responsible for actively unfolding the protein substrate in a process that requires ATP [9,10]. In contrast, the 20S proteasome does not require ATP for activity [11].

The proteolytic activity of the proteasome resides within the inner β-rings where each of the β1, β2 and β5 subunits possess specific activities—primarily peptidyglutamyl peptide hydrolyzing-like (PGPH or caspase-like), trypsin-like (TRP), or chymotrypsin-like (CTL), respectively [12]. The CTL activity cleaves primarily after hydrophobic residues and studies have suggested it may be involved in the degradation of ubiquitin-tagged IκB, an inhibitor protein of the pro-inflammatory transcription factor NF-κB [13,14]. The PGPH-like activity favors cleavage after acidic amino acid residues, and the TRP-like activity cleaves after basic amino acids and plays an important role in generating peptides for antigen presentation by major histocompatibility complex (MHC) class I molecules [15,16].

Immunoproteasome subunits β1i, β2i, and β5i are induced to replace standard β subunits under specific physiologic conditions (e.g., IFN-γ release increases β1i and β5i expression [17,18]). Expression of these subunits results in proteasomes that enhance the immune response by creating a larger repertoire of peptides available for MHC class I presentation [15,19].

Although previous studies have investigated proteolytic activity in adult tissue [20,21,22] or have studied proteasomes during advanced aging [23], to date there is little data available on activity during earlier developmental stages. Despite evidence that proteasomes play an important role in the control of intestinal homeostasis in the adult [24], no data is available on the intestine of infants. The infant intestinal immune response is unique, as recent evidence suggests that decreased immune activation in early neonatal life is developmentally essential to permit normal colonization with commensal organisms [25]. As proteasomes play an important role in the host immune response, in this study we hypothesized that intestinal 26S proteasomes are developmentally regulated. These studies are difficult to accomplish in human patients; however, earlier studies have used rodent models to investigate proteasome development in other tissues [26].

An affinity-chromatography approach was used to purify functional 26S proteasomes from rat intestine from several time points ranging from birth to post-weaning. Intestinal samples were compared to liver and spleen, since proteasome developmental expression in these organs has been previously reported [26]. These studies demonstrated a developmental pattern to 26S proteasome subunit expression and activity, with decreased activity at birth that increased at weaning. β catalytic subunits, structural subunits, and regulatory subunits were present in early developmental stages, while expression of immunoproteasome subunits did not appear until later time points. Taken together, the low initial 26S proteasome expression and activity that then increases during development is compatible with the existence of a hyporesponsive adaptive immune response in the immediate newborn period, reported by others, and considered necessary at this developmental stage until microbial colonization has been established.

## 2. Results and Discussion

### 2.1. CTL-, TRP-, and PGPH-Like Activity Increases from Birth to Post-Weaning

CTL-, TRP-, and PGPH-like activity were measured from rat intestine, with liver and spleen compared as controls over several different developmental time points ranging from birth (0 days old, DO), pre-weaning (one week old, WO), weaning (3 WO), and post weaning (5 WO). These activities were present in all three tissues, but increased over time from birth to post-weaning.

**Figure 1:**
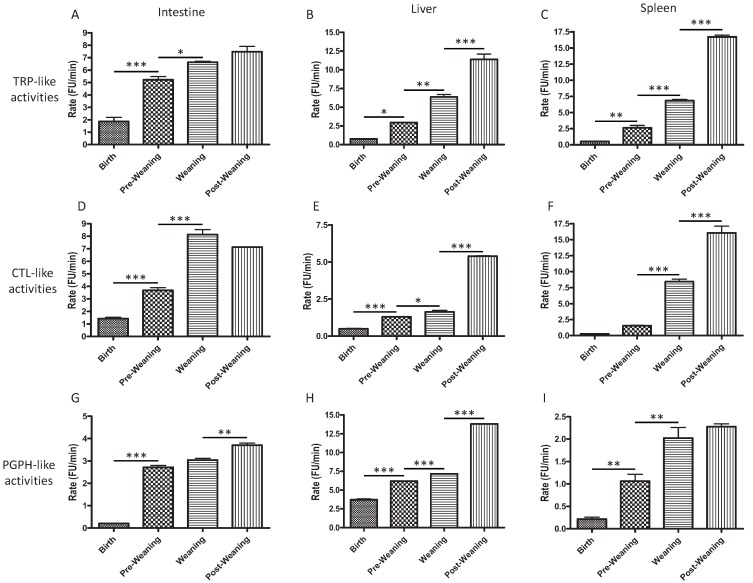
TRP- (**A**,**B**,**C**), CTL- (**D**,**E**,**F**), and PGPH-like (**G**,**H**,**I**) activities of 26S proteasomes isolated from intestine (**A**,**D**,**G**), liver (**B**,**E**,**H**) and spleen (**C**,**F**,**I**). Proteasomes were isolated using affinity purification at time points chosen from birth (0 days old, DO), pre-weaning (one week old, WO), weaning (3 WO), and post-weaning (5 WO) and assayed for catalytic activity as described in methods. 25 µg of proteasomes were used for all activity assays. * *p* < 0.05, ** *p* < 0.01, *** *p* < 0.001.

Corresponding to the β2 and β5 subunit expression, TRP-like activity, which is known to play an important role in antigen presentation, as well as CTL activity, which may be involved in the regulation of inflammatory pathways through its function of IκB degradation [27], significantly increased at weaning in the intestine, liver and spleen relative to pre-weaning (Figure 1A–C for TRP-like activity and Figure 1D–F for CTL-like activity). In the intestine, PGPH-like activity increased at post-weaning (Figure 1G, *p* < 0.01). PGPH-like activity increased in the liver (Figure 1H, *p* < 0.001) and spleen (Figure 1I, *p* < 0.01) at weaning. However, PGPH-like activity remained fairly low in spleen, consistent with its proteolytic cleavage after acidic residues and therefore, its lack of MHC-presentation function. Our results are consistent with those of Abramova *et al*. (2005) demonstrating increased CTL activity with age in rat liver and spleen (adult rats compared to 1 DO rats) [26]. It is also consistent with reports of less efficient antigen processing and presentation in infants, resulting in a reduced ability to detect and respond to microorganisms compared to adults [28].

### 2.2. Core Proteasome Subunit Expression Increases from Birth to Post-Weaning

The CTL and TRP-like activities of proteasomes contribute to the immune response by generating hydrophobic and basic peptides (epitopes) for antigen presentation by major histocompatibility complex (MHC) class I molecules [15,16]. MHC class I molecules typically display ligands containing either basic or hydrophobic residues on the C-terminus. The immune system can modify the activity of the proteasome in sites of inflammation by replacing proteasomal active site subunits and regulators through an inflammatory cytokine-mediated induction, e.g., with IFN-γ and TNF-α [15,29]. By altering the cleavage preferences of the proteasome, immunoproteasome expression affects pathogen-specific CD8 T cell responses [30]. Therefore, the expression of proteasome and immunoproteasome subunits was investigated.

**Figure 2:**
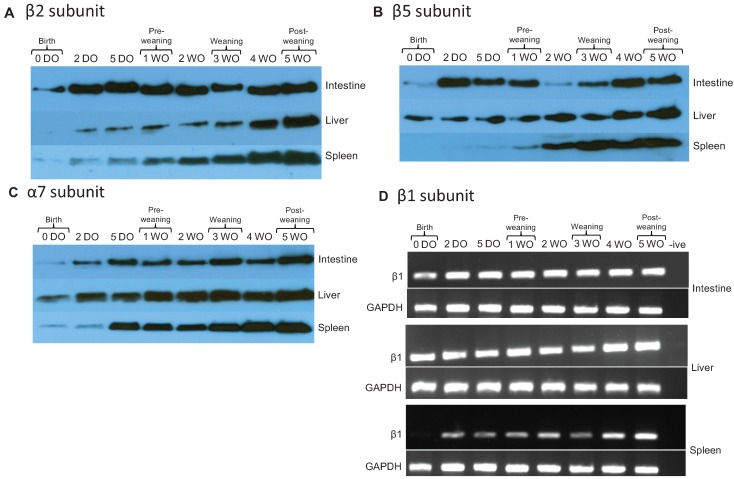
Expression of “core” 26S proteasome subunits during development in rat. Expression of β2 (**A**), β5 (**B**), and α7 (**C**) were measured by Western blot analysis. 26S proteasomes were affinity-purified and the concentrations of purified protein isolated from intestine, liver, and spleen tissue were measured using the BCA (bicinchoninic acid) protein quantification kit (Pierce, Rockford, IL, USA). The presence of the β1 subunit (and housekeeping gene GAPDH) were assessed by RT-PCR using tissues homogenized with TRIzol reagent (**D**). The data shown are representative of three independent experiments.

Proteasome subunit expression was measured from affinity purified 26S proteasomes from the rat intestine, liver, and spleen at time points from birth to post-weaning (0 DO to 5 WO). Since all of the proteolytic activity of the proteasome resides within the inner β-rings of the proteasome, Western blot was used to measure representative β (specifically β2 and β5) and α (specifically α7) subunits. All subunits were present at birth (0 DO) in the intestine. β2 subunit expression increased in the intestine and spleen from birth (0 DO) to post-weaning (5 WO), and in the liver from 2 DO to post-weaning (5 WO; Figure 2A). The β5 subunit expression increased in the intestine and liver from birth (0 DO) to post-weaning (5 WO), and in the spleen from 2 DO to post-weaning (5 WO; Figure 2B). The α7 subunit was detected in the intestine, liver, and spleen from birth. Due to difficulties encountered with antibody availability we were unable to perform immunoblot analysis for the β1 subunit, however analysis by RT-PCR of rat tissues revealed that mRNA for this subunit was present at all time points (Figure 2D), consistent with the functional assays in Figure 1 indicating that β1 enzymatic activity (PGPH or caspase-like activity) was present at early time points.

Our results demonstrate differences in the expression of core proteasome subunits from proteasomes isolated from intestine, liver, and spleen. Consistent with our findings, a study by Kuckelkorn *et al*. (2002) found that proteasomes isolated from a variety of tissues from adult mice, including the intestine and liver, expressed characteristic compositions of α and β chain subunits and produced distinct peptide fragments (in terms of quality and quantity) [31]. They proposed that proteasomes have tissue-specific antigen processing capabilities that may control organ-specific immune responses [31]. Our results in the intestine specifically demonstrate expression of all subunits at low levels at birth, and increasing over time. This pattern is also consistent with other reports of decreased immune responses at birth but increasing at later developmental stages [25,32].

### 2.3. Expression of 19S Proteasome ATPase Subunit in Rat Intestine, Liver, and Spleen Increases over Time

**Figure 3:**
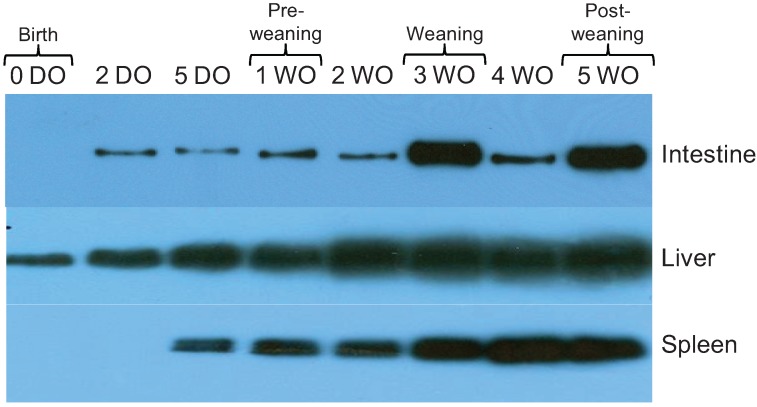
Western blot analysis of affinity purified 19S proteasome subunit Rpt5. The concentrations of purified proteins isolated from intestine, liver, and spleen tissue were measured using the BCA protein quantification kit (Pierce, Rockford, IL, USA). The data shown are representative of three independent experiments.

The 19S subunit is composed of a lid and a base, and the base contains a hexameric ring of ATPases of 6 subunits (Rpt1-6) that are responsible for unfolding the protein substrate [9,10]. Rpt5 plays a role in triggering the opening of the proteasome channel and in tethering ubiquitinated proteins to the proteasome [10,33,34]. The Rpt5 subunit was present at birth only in the liver, and appeared shortly after birth in intestine (2 DO) and spleen (5 DO) (Figure 3). Similar to the 20S proteasome subunits, Rpt5 levels increased over time.

Although expression in developing intestine has not been previously reported, post-natal 19S proteasomal subunit composition has previously been investigated in liver and spleen tissues of developing rats [26]. Abramova *et al*. found that the 19S proteasomal subunit Rpt6 was present in liver and spleen tissues at all stages of postnatal rat development [26]. This is different from our results with Rpt5. It has been shown that in mouse myoblasts and myotubes different Rpt ATPase subunits of the 19S complex display different regulatory effects during development, and display an asymmetry of function in acceptance of peptides [35]. By time of weaning, we demonstrate that Rpt5 expression had increased markedly in intestine.

### 2.4. Immunoproteasomes are Developmentally Regulated and Appear at Later Time Points

**Figure 4:**
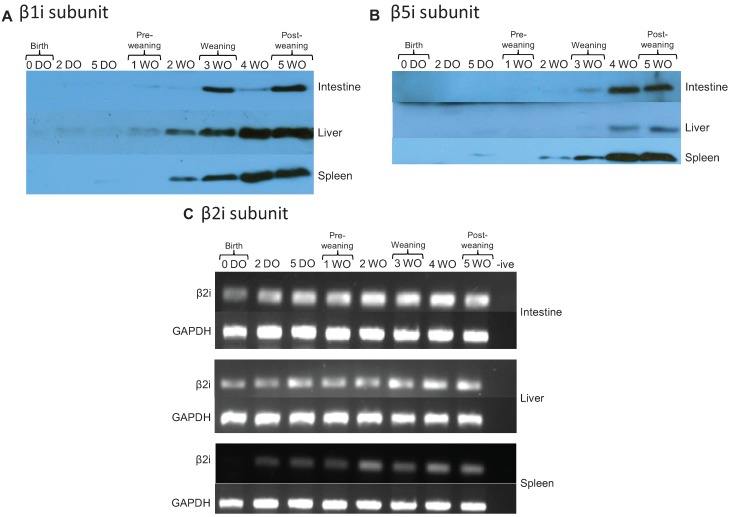
Expression of immunoproteasome subunits during development. Expression of β1i (**A**) and β5i (**B**) were measured by Western blot analysis. Proteasomes were affinity-purified and the concentrations of purified protein isolated from intestine, liver, and spleen tissue were measured using the BCA protein quantification method. The presence of the β2i subunit (and housekeeping gene GAPDH) was assessed by RT-PCR using tissues homogenized with TRIzol reagent (**C**). The data shown are representative of three independent experiments.

The subunits of immunoproteasomes (β1i, β2i, and β5i) have different catalytic profiles with less PGPH-like and more CTL/TRP-like activity, and are believed to play an important role in enhancing the immune response by creating a larger repertoire of peptides for MHC class I presentation [15,19]. The developmental expression of immunoproteasome subunits was determined in rat intestine, liver, and spleen. Immunoblotting analysis of β1i and β5i demonstrated that these subunits increased over time and were not present at birth. The expression of β1i showed only minimal expression at early time points. Significant expression was not seen until weaning (three weeks) in intestine and two weeks in liver and spleen (Figure 4A). The β5i subunit was detected in the intestine at weaning (week 3) and in the liver at post-weaning (week 4; Figure 4B). β5i expression was the highest in spleen tissue, with expression first detected in pre-weaning (5 DO) rats and increasing over time. Due to difficulties encountered with antibody availability we were unable to perform immunoblot analysis for the β2i subunit, however analysis by RT-PCR revealed that mRNA for this subunit was present in intestine and liver at all time points (Figure 4C). Collectively, these data demonstrate that the immunoproteasome subunits also follow a developmental pattern and increase over time, however unlike core proteasomes at birth, the immunoproteasomes were not revealed by the methods applied. These results are different from those found by others in other tissues. Specifically, Sharova *et al*. demonstrated immune proteasomes in the spleen and liver of Wister rats in the fetal period and at birth [36], which highlights the uniqueness of the intestine, and its need to allow rapid microbial colonization immediately after birth [36,37].

Immunoproteasome subunit composition (β1i) has previously been investigated in liver and spleen tissues of developing rats [26]. Abramova *et al*. found β1i was expressed in the rat spleen by day 9 and in the liver by day 23 (of postnatal development) [26]. In contrast, our data in Figure 3 shows expression by day 14 in liver. These differences between the results of Abramova *et al.* may be partially explained by differences in proteasome purification methods as our study used affinity chromatography, whereas their study used ammonium sulfate precipitation and intentionally focused on partially enriched, rather than highly purified, proteasome preparations [26].

Our study is primarily interested in intestine, an organ that has remained largely undescribed with respect to proteasome expression during postnatal development. In studies of disease, Visekruna *et al*. (2006) have shown that inflamed mucosa of Crohn’s disease patients, and to a lesser extent, ulcerative colitis patients, exhibit increased expression of immunoproteasomes [24], consistent with an increased immune and inflammatory response to commensal flora as part of the pathophysiology of this disease. Correspondingly, proteasomes isolated from the intestine and colon of Crohn’s disease and ulcerative colitis patients have been shown to display different subunit composition and proteolytic activity. Moreover, β5i (LMP7) was more highly expressed in inflamed mucosa from Crohn’s disease patients. Our studies have instead focused on the normal development of 26S proteasomes in a non-diseased state. Our data demonstrates decreased 26S proteasome activity at early time points, delayed expression of immunoproteasomes, with corresponding developmental progression in subunit expression and increase in activity over time. This progression is consistent with studies demonstrating hyporesponsiveness of other aspects of the adaptive immune system immediately after birth. This initial decrease in immune response results in a necessary paradox in infants, of increased susceptibility to infection to allow critical establishment of normal colonizing microbiota after birth.

## 3. Experimental

### 3.1. Ethics Statement

This study was carried out in strict accordance with the recommendations in the Guide for the Care and Use of Laboratory Animals of the National Institutes of Health. All animal work was conducted under the animal protocol No. 71452 and was approved by the University of Chicago Institutional Animal Care and Use Committee (IACUC). All efforts were made to minimize suffering. Each animal was humanely euthanized with CO_2_ or isoflurane overdose followed by decapitation.

### 3.2. Animals

Timed-pregnant Sprague-Dawley rats (Harlan Laboratories, Madison, WI, USA) were weaned 3 weeks after birth and sacrificed at different development stages (from birth to 5 weeks after birth). Liver, spleen and intestine were obtained after dissection. Tissues were harvested from at least three animals at each time point and data were obtained from 3 separate experiments.

### 3.3. Antibodies, Chemicals and DNA Constructs

Antibodies (Anti-β2 (PW9300), anti-β5 (PW8895), anti-β1i (PW8840), anti-β5i (PW8845), anti-Rpt5 (PW8770)), proteasome fluorogenic substrates (*N*-Succinyl-Leu-Leu-Val-Tyr-7-amino-4-methylcoumarin (Suc-LLVY-AMC), Z-Leu-Leu-Glu 7-amino-4-methylcoumarin (Z-LLE-AMC), and Benzyloxycarbonyl-Leu-Arg-Arg-7-amino-4-methylcoumarin (Boc-LRR-AMC) were purchased from Enzo Life Science (Farmingdale, NY, USA). Isopropylthio-β-galactoside (IPTG) was obtained from Invitrogen Life Technologies (Burlington, ON, USA). Nickel nitriloacetic acid (Ni-NTA) agarose was purchased from Qiagen (Toronto, ON, Canada), Glutathione-Agarose was obtained from Sigma Aldrich (Oakville, ON, Canada). *Escherichia coli* BL21 (DE3) competent cells were purchased from Stratagene (Santa Clara, CA, USA) and Sigma Aldrich. ATP was obtained from Amersham Biosciences (Piscataway, NJ, USA). All primers used for reverse transcriptase PCR were purchased from Sigma Aldrich.

### 3.4. Expression and Purification of pET15b-mUIM-2

A mUIM-2 motif (amino acids 244–351) was subcloned into pET15b, a HIS-tag expression vector, with *Pvu* II/*Bam*H I to create pET15b-mUIM-2, kindly provided to us by Lenore K. Beitel [38]. pET15b-mUIM-2 plasmid was transformed into *Escherichia coli* BL21(DE3) heat shock competent cells and bacteria were grown in Luria-Bertani (LB) broth, Lennox, with ampicillin (100 µg/mL; Fisher BioReagents, Ottawa, ON, Canada) in a New Brunswick Scientific Innova 4000 incubator shaker (37 °C) (Enfield, CT, USA) to an optical density (O.D.) of 0.6 (A600 nm). The culture was then induced with 0.25 mM IPTG, and after 16 h at 37 °C, bacteria were harvested by centrifugation at 3000× *g* for 15 min at 4 °C. Cell pellets were resuspended in buffer A (50 mM HEPES, pH 7.5, 300 mM NaCl, 25 mM imidazole; Fisher Scientific, Ottawa, ON, Canada), and lysed by sonication. Lysed cells were then centrifuged at 30,000× *g* for 30 min at 4 °C, the supernatant was loaded onto a Ni-NTA column, and the His_6_ tagged mUIM-2 was eluted with a gradient from 25 mM to 500 mM imidazole. The fractions eluted were then analyzed with 12% SDS-PAGE gels followed by Coomassie Blue G-250 (Fisher Scientific, Ottawa, ON, Canada) staining (data not shown) [39]. Fractions containing pure mUIM-2 were pooled and subsequently concentrated with Millipore Amicon Ultra centrifugal devices (10,000 MWCO).

### 3.5. Expression and Purification of pGEX-2T-UBL

The genes encoding residues 1 ± 87 of the Ubl domain of HHR23A were cloned into a pGEX-2T expression system (Pharmacia, Stockholm, Sweden) to create pGEX-2T-UBL, kindly provided to us by Juli Feigon [40]. pGEX-2T-UBL plasmid was expressed by transformation of the plasmid into *Escherichia coli* BL21(DE3)ccc cells. Cells were grown in LB broth with ampicillin (100 µg/mL) in an Innova 4000 incubator shaker (250 rpm, 37 °C) to an O.D of 0.6 (A600 nm), and induced with 0.25 mM IPTG. After 4 h at 37 °C, the cells were harvested by centrifugation at 3000× *g* at 4 °C for 15 min. The cell pellets were then resuspended in purification buffer (50 mM HEPES, pH 7.5, 5 mM MgCl_2_, 10% glycerol; Fisher Scientific) and lysed by sonication. The lysed cells were then centrifuged at 30,000× *g* at 4 °C for 30 min, and the supernatant was loaded onto glutathione-agarose beads, rocking at 4 °C for 2 h. The agarose beads were centrifuged at 500× *g* for 2 min, supernatant was removed, and beads were washed with 10 column volumes of purification buffer. Sample purity of the fusion protein was confirmed by resolving the GST-UBL beads on a 12% SDS-PAGE gel and visualizing by Coomassie Blue G-250 (Fisher Scientific, Ottawa, ON, Canada) staining (data not shown) [39].

### 3.6. 26S Proteasome Purification from Rat Tissues

At each time point, tissue from three rats was pooled and subsequent experiments carried out with the purified 26S proteasomes (repeated in triplicate—total of 9 tissue samples analyzed per time point for activity assays). 26S proteasomes from rat tissue were purified according to Scanlon *et al.* [38] and Besche *et al.* [41] with some modifications. In brief, 40 mg to 750 mg of rat tissues (intestines, livers, or spleens) were homogenized in purification buffer (50 mM HEPES, pH 7.5, 5 mM MgCl_2_, 10% glycerol) supplemented with 2 mM ATP and protease inhibitor cocktail (Roche, Laval, QC, USA) (Note: for intestine, 3 times more of the protease inhibitor cocktail was used), and the homogenized solution was centrifuged at 21,100× *g* for 1 h at 4 °C. Supernatants were then loaded onto the GST-UBL beads. The same amount of GST-UBL protein and GST beads were used for each sample, and the GST-UBL beads served as the affinity matrix. The suspension was rotated at 4 °C for 4 h in a 15 mL conical tube, and the beads were then washed with 10 volumes of purification buffer containing 2 mM ATP. 26S proteasome bound to the GST-UBL matrix was eluted by using 20-fold molar excess of purified mUIM-2-HIS (to completely elute 26S proteasomes), which was left under continuous mixing overnight at 4 °C. 500 μL of Ni-NTA agarose beads were added to the eluate and incubated at 4 °C for 2 h to remove excess mUIM-2-HIS. The supernatants were then collected by centrifuging at 500× *g* for 3 min, and a BCA assay (Pierce, Nepean, ON, Canada) was performed to determine protein concentration of the samples. Average yield was 500 µg total protein per pooled sample.

### 3.7. Proteasome Activity Assay

Proteasome activity was monitored on a Varian Cary Eclipse fluorescence spectrophotometer by using a fluorogenic substrate assay. In summary, 25 μg of affinity purified 26S proteasome (0.17 µg/mL final concentration in assay) was incubated in the reaction buffer (25 mM HEPES/pH 7.6, 10 mM EDTA, 0.03% SDS) supplemented with either 65 μM of Suc-LLVY-AMC, Z-LLE-AMC, or Boc-LRR-AMC, 5 mM MgCl_2_ and 2 mM ATP were also present as carry over from the purification buffer (see Section 3.6). Fluorescence generated from free AMC was monitored every minute for 30 min over the linear range of the reaction (Excitation: 380 nm, emission: 460 nm), and proteasome activity was determined by calculating the rate of free AMC released over time using the Varian Cary Eclipse kinetics application software. Experiments were performed in triplicate with 96-well half area black polystyrene plates (Corning Incorporated, Corning, NY, USA).

### 3.8. Western Blot Analysis

Affinity purified proteasomes from rat tissues were separated by SDS-PAGE (12% gels) for Western blot analysis with specific antibodies for Anti-β2, anti-β5, anti-β1i, anti-β5i, and Rpt5. Following SDS-PAGE, samples were transferred to Immobilon polyvinylidene difluoride (PVDF) membrane (Millipore, Bedford, MA, USA) at 200 mA in transfer buffer (50 mM Tris, 40 mM glycine, 20% (*v/v*) methanol; Fisher Scientific) for 60–90 min. Membranes were blocked for at least 1 h with 5% (*w/v*) non-fat milk, and incubated with the appropriate primary antibodies (1:2000 dilution) overnight at 4 °C, followed by 3 washes with Tris buffered saline with Tween 20 (TBST; Tween 20, Bio Basic Inc., Markham, ON, Canada). Horseradish peroxidase (HRP)-conjugated secondary antibodies were added (1:10,000 dilution) and membranes incubated for 1 h at room temperature. After 3 washes with TBST, immunoreactive bands were visualized by chemiluminescence using Supersignal West Pico ECL reagents (Thermo Scientific, Nepean, ON, Canada) followed by exposure of the membranes to autoradiographic film (VWR, Mississauga, ON, Canada).

### 3.9. RNA Extraction and RT-PCR

Around 100 mg of rat tissues were homogenized in 1 mL of TRIzol Reagent with a homogenizer (VWR) and total RNA was purified according to the manufacturer’s instructions (Invitrogen Life Technologies, Burlington, ON, Canada). cDNA was reverse transcribed from 3 μg of RNA using the oligo (dT) primers and SuperScript III reverse transcriptase (Invitrogen Life Technologies). Each cDNA sample was incubated in 50 μl of reaction containing *Taq* polymerase (Thermo Scientific, Waltham, MA, USA), 4 mM MgCl_2_, 5% (*v/v*) DMSO, 0.2 mM dNTP’s, and the following primers: β2i: forward, 5'-GCACTGTTGGAGGACCGGTTCCA-3'; reverse, 5'-AGTGATCACACAGGCATCCACA-3'; β1: forward, 5'-GACAAAACAGTAATTGGCTGC-3'; reverse, 5'-CGGCTGCAGCATGGCA CTTGC-3'. The GAPDH housekeeping gene was used as the positive control: forward, 5'-TGACAACTTTGGCATCGTGG-3'; reverse, 5'-TACTCCTTGGAGGCCATGT-3'. For all reactions, the thermocycler was set for 35 cycles at an annealing temperature of 58 °C. The final PCR product were separated on 1.5% (*w/v*) agarose gel containing 0.1 μg/mL ethidium bromide, and visualized using the Alphamager EC (Alpha Innotech, Santa Clara, CA, USA) system.

### 3.10. Statistical Analysis

Data are expressed as mean ± SEM. Statistical significance was evaluated by one-away ANOVA with Bonferroni correction. Graphs represent mean of value from three independent experiments. *p* < 0.05 was considered significant.

## 4. Conclusions

Due to their key biological functions, proteasomes must function in a developmentally appropriate manner. However, to date few studies have investigated proteasomal activity in the early stages of life, and none have focused on the intestine, a site important as the largest human reservoir of colonizing microbiota and a key immune organ. This work investigated developmental progression of 26S proteasome complexity and function in rat intestine, as well as liver and spleen tissues from rats ranging from birth to 5 weeks old. We found that the protein expression of subunits associated with the core 20S barrel (β2, β5, and α7 subunits) and regulatory subunit (Rpt5) were present in early developmental stages. We saw a gradual developmental progression of 26S proteasome structure with the increased expression of β1i and β5i immunoproteasome subunits over time. Incorporation of immunosubunits alters the core proteasome cleavage preferences and enhances the generation of antigenic peptides [42]. In addition to changes in subunit expression, our study demonstrated a developmental pattern to 26S proteasome activity, with increased catalytic activity around the time of weaning. Many changes occur at weaning, including alteration in food substrate, separation from mother, and change in intestinal microbiota [43]. This study suggests decreased proteasome functions at birth which may limit responses to microorganisms as commensal microbial patterns are being established, followed by appearance of more complex forms of the proteasome responsible for the tight control of cellular protein homeostasis, inflammation, and immune function which are required later in the course of development.

## References

[1] Claud E.C., Savidge T., Walker W.A. (2003). Modulation of human intestinal epithelial cell IL-8 secretion by human milk factors. Pediatr. Res..

[2] Nanthakumar N.N., Fusunyan R.D., Sanderson I., Walker W.A. (2000). Inflammation in the developing human intestine: A possible pathophysiologic contribution to necrotizing enterocolitis. Proc. Natl. Acad. Sci. USA.

[3] Goldberg A.L. (2003). Protein degradation and protection against misfolded or damaged proteins. Nature.

[4] Abramova E.B., Sharova N.P., Karpov V.L. (2002). The proteasome: destroy to live. Mol. Biol..

[5] Fietta P., Delsante G. (2010). Proteasomes and immunoproteasomes. Riv. Biol..

[6] Dahlmann B., Ruppert T., Kuehn L., Merforth S., Kloetzel P.M. (2000). Different proteasome subtypes in a single tissue exhibit different enzymatic properties. J. Mol. Biol..

[7] Drews O., Wildgruber R., Zong C., Sukop U., Nissum M., Weber G., Gomes A.V., Ping P. (2007). Mammalian proteasome subpopulations with distinct molecular compositions and proteolytic activities. Mol. Cell. Proteomics.

[8] Kloetzel P.M., Ossendorp F. (2004). Proteasome and peptidase function in MHC-class-I-mediated antigen presentation. Curr. Opin. Immunol..

[9] Finley D. (2009). Recognition and processing of ubiquitin-protein conjugates by the proteasome. Annu. Rev. Biochem..

[10] Pickart C.M., Cohen R.E. (2004). Proteasomes and their kin: Proteases in the machine age. Nat. Rev. Mol. Cell Biol..

[11] Davies K.J. (2001). Degradation of oxidized proteins by the 20S proteasome. Biochimie.

[12] Pickering A.M., Davies K.J. (2012). Degradation of damaged proteins: The main function of the 20S proteasome. Prog. Mol. Biol. Transl. Sci..

[13] Traenckner E.B., Wilk S., Baeuerle P.A. (1994). A proteasome inhibitor prevents activation of NF-kappa B and stabilizes a newly phosphorylated form of I kappa B-alpha that is still bound to NF-kappa B. EMBO J..

[14] Petrof E.O., Kojima K., Ropeleski M.J., Musch M.W., Tao Y., de Simone C., Chang E.B. (2004). Probiotics inhibit nuclear factor-kappaB and induce heat shock proteins in colonic epithelial cells through proteasome inhibition. Gastroenterology.

[15] Khan S., van den Broek M., Schwarz K., de Giuli R., Diener P.A., Groettrup M. (2001). Immunoproteasomes largely replace constitutive proteasomes during an antiviral and antibacterial immune response in the liver. J. Immunol..

[16] Groettrup M., Kirk C.J., Basler M. (2010). Proteasomes in immune cells: More than peptide producers?. Nat. Rev. Immunol..

[17] Brooks P., Murray R.Z., Mason G.G., Hendil K.B., Rivett A.J. (2000). Association of immunoproteasomes with the endoplasmic reticulum. Biochem. J..

[18] Gaczynska M., Rock K.L., Goldberg A.L. (1993). Gamma-interferon and expression of MHC genes regulate peptide hydrolysis by proteasomes. Nature.

[19] Kremer M., Henn A., Kolb C., Basler M., Moebius J., Guillaume B., Leist M., van den Eynde B.J., Groettrup M. (2010). Reduced immunoproteasome formation and accumulation of immunoproteasomal precursors in the brains of lymphocytic choriomeningitis virus-infected mice. J. Immunol..

[20] Shibatani T., Nazir M., Ward W.F. (1996). Alteration of rat liver 20S proteasome activities by age and food restriction. J. Gerontol. A Biol. Sci. Med. Sci..

[21] Noda C., Tanahashi N., Shimbara N., Hendil K.B., Tanaka K. (2000). Tissue distribution of constitutive proteasomes, immunoproteasomes, and PA28 in rats. Biochem. Biophys. Res. Commun..

[22] Farout L., Lamare M.C., Cardozo C., Harrisson M., Briand Y., Briand M. (2000). Distribution of proteasomes and of the five proteolytic activities in rat tissues. Arch. Biochem. Biophys..

[23] Chondrogianni N., Gonos E.S. (2005). Proteasome dysfunction in mammalian aging: Steps and factors involved. Exp. Gerontol..

[24] Visekruna A., Joeris T., Seidel D., Kroesen A., Loddenkemper C., Zeitz M., Kaufmann S.H., Schmidt-Ullrich R., Steinhoff U. (2006). Proteasome-mediated degradation of IκBα and processing of p105 in Crohn disease and ulcerative colitis. J. Clin. Invest..

[25] Elahi S., Ertelt J.M., Kinder J.M., Jiang T.T., Zhang X., Xin L., Chaturvedi V., Strong B.S., Qualls J.E., Steinbrecher K.A. (2013). Immunosuppressive CD71^+^ erythroid cells compromise neonatal host defence against infection. Nature.

[26] Abramova E.B., Astakhova T.M., Sharova N.P. (2005). Changes in proteasome activity and subunit composition during postnatal development of rat. Ontogenez.

[27] Chen Z.J. (2005). Ubiquitin signalling in the NF-kappaB pathway. Nat. Cell. Biol..

[28] Hunter C.J., Upperman J.S., Ford H.R., Camerini V. (2008). Understanding the susceptibility of the premature infant to necrotizing enterocolitis (NEC). Pediatr. Res..

[29] Groettrup M., Soza A., Kuckelkorn U., Kloetzel P.M. (1996). Peptide antigen production by the proteasome: Complexity provides efficiency. Immunol. Today.

[30] Sijts E.J., Kloetzel P.M. (2011). The role of the proteasome in the generation of MHC class I ligands and immune responses. Cell. Mol. Life Sci..

[31] Kuckelkorn U., Ruppert T., Strehl B., Jungblut P.R., Zimny-Arndt U., Lamer S., Prinz I., Drung I., Kloetzel P.M., Kaufmann S.H. (2002). Link between organ-specific antigen processing by 20S proteasomes and CD8^+^ T cell-mediated autoimmunity. J. Exp. Med..

[32] Chassin C., Kocur M., Pott J., Duerr C.U., Gutle D., Lotz M., Hornef M.W. (2010). miR-146a mediates protective innate immune tolerance in the neonate intestine. Cell. Host Microbe.

[33] Smith D.M., Chang S.C., Park S., Finley D., Cheng Y., Goldberg A.L. (2007). Docking of the proteasomal ATPases’ carboxyl termini in the 20S proteasome’s alpha ring opens the gate for substrate entry. Mol. Cell..

[34] Kruger E., Kuckelkorn U., Sijts A., Kloetzel P.M. (2003). The components of the proteasome system and their role in MHC class I antigen processing. Rev. Physiol. Biochem. Pharmacol..

[35] Wang X.H., Zhang L., Mitch W.E., LeDoux J.M., Hu J., Du J. (2010). Caspase-3 cleaves specific 19S proteasome subunits in skeletal muscle stimulating proteasome activity. J. Biol. Chem..

[36] Sharova N.P., Zakharova L.A., Astakhova T.M., Karpova Y.D., Melnikova V.I., Dmitrieva S.B., Lyupina Y.V., Erokhov P.A. (2009). New approach to study of T cellular immunity development: Parallel investigation of lymphoid organ formation and changes in immune proteasome amount in rat early ontogenesis. Cell. Immunol..

[37] Karpova Y.D., Lyupina Y.V., Astakhova T.M., Stepanova A.A., Erokhov P.A., Abramova E.B., Sharova N.P. (2013). Immune proteasomes in the development of the rat immune system. Russ. J. Bioorg. Chem..

[38] Scanlon T.C., Gottlieb B., Durcan T.M., Fon E.A., Beitel L.K., Trifiro M.A. (2009). Isolation of human proteasomes and putative proteasome-interacting proteins using a novel affinity chromatography method. Exp. Cell. Res..

[39] Claud E.C., McDonald J.A.K., He S.-M., Yu Y., Duong L., Sun J., Petrof E.O. (2014).

[40] Mueller T.D., Feigon J. (2003). Structural determinants for the binding of ubiquitin-like domains to the proteasome. EMBO J..

[41] Besche H.C., Haas W., Gygi S.P., Goldberg A.L. (2009). Isolation of mammalian 26S proteasomes and p97/VCP complexes using the ubiquitin-like domain from HHR23B reveals novel proteasome-associated proteins. Biochemistry.

[42] Sijts A., Sun Y., Janek K., Kral S., Paschen A., Schadendorf D., Kloetzel P.M. (2002). The role of the proteasome activator PA28 in MHC class I antigen processing. Mol. Immunol..

[43] Amarri S., Benatti F., Callegari M.L., Shahkhalili Y., Chauffard F., Rochat F., Acheson K.J., Hager C., Benyacoub J., Galli E. (2006). Changes of gut microbiota and immune markers during the complementary feeding period in healthy breast-fed infants. J. Pediatr. Gastroenterol. Nutr..

